# Supplementary material for: Understanding European integration with bipartite networks of comparative advantage
Source: PNAS Nexus. 2022 Nov 27;1(5):pgac262. doi: 10.1093/pnasnexus/pgac262 (PMC9802098; doi:10.1093/pnasnexus/pgac262)
Supplement: pgac262_Supplemental_File [file pgac262_supplemental_file.pdf]

Supplementary Materials

Understanding European Integration with Bipartite Networks of Comparative Advantage

Riccardo Di Clemente,[1,2,*] Balázs Lengyel, [3,4,‡] Lars F. Andersson[5], Rikard Eriksson[6]

[1]The Alan Turing Institute, London, NW1 2DB, United Kingdom.
[2]Exeter University, Department of Computer Science, Exeter, EX4 EPY, United Kingdom.
[3]Agglomeration and Social Networks Lendület Research Group, Centre for Economic and Regional Studies, Eötvös Loránd Research Network, Budapest, 1097, Hungary.
[4]Corvinus University of Budapest, Budapest, 1093, Hungary.
[5]Umeå University, Department of Economic history, SE-90187, Umeå Sweden.
[6]Umeå University, Department of Geography, SE-90187, Umeå, Sweden.
[*]To whom correspondence should be addressed E-mail: r.di-clemente@exeter.ac.uk
[‡]To whom correspondence should be addressed E-mail: lengyel.balazs@krtk.hu

S1 Supplementary Material 1: Z-score trends of sectors

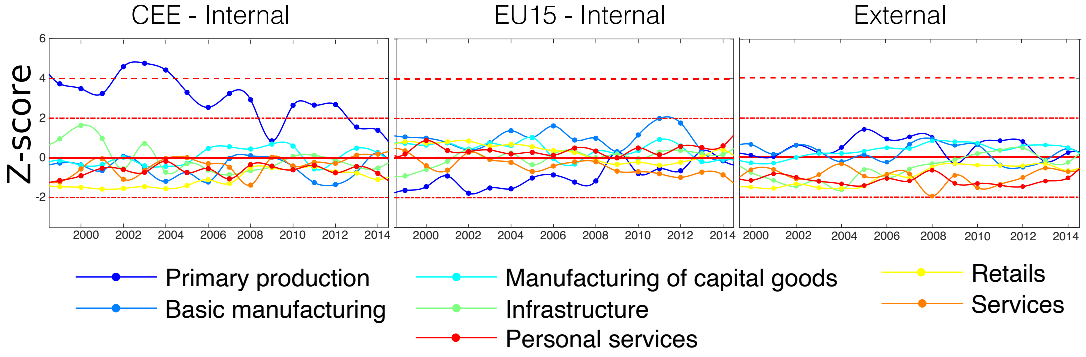

FigureS1.1: Z-scores of co-specialization motifs decomposed into main sectors.

S2 Supplementary Material 2: Definitions, descriptive and correlations of country-industry variables

Table S2.1: Definition of regression variables

Variable	Definition
Overall	z-score of co-specialization motifs without country limitations (standardized)
Internal	z-score of co-specialization motifs within groups of EU15 or CEE countries (standardized)
External	z-score of co-specialization motifs across groups of EU15 and CEE countries (standardized)
Entry	Dummy=1 if year >= 2004
CEE	Central and Eastern European Countries
Recession	Dummy=1 if year = 2008 or 2009
GFC	Gross capital formation (standardized)
EMP	Employment (standardized)

Table S2.2: Summary statistics of regression variables

Variable	Obs	Mean	St.Dev.	Min	Max
Overall	9,555	0.001	0.999	-2.156	2.852
Internal	9,555	-0.001	0.993	-1.691	3.912
External	9,555	0.002	0.991	-1.863	6.016
Entry	11,160	0.733	0.442	0	1
CEE	11,160	0.292	0.454	0	1
Recession	11,160	0.067	0.249	0	1
GFC	8,572	0.261	0.772	-8.309	6.112
EMP	10,977	0.004	1.015	-0.496	9.935

Table S2.3: Pair-wise Pearson correlation of regression variables

#	Variable	1	2	3	4	5	6	7
1	Overall							
2	Internal	0.888						
3	External	0.709	0.345					
4	Entry	0.002	0.011	-0.009				
5	CEE	0.002	0.102	-0.023	-0.000			
6	Recession	0.001	0.003	-0.003	0.161	-0.000		
7	GFC	0.023	0.031	-0.014	0.440	-0.057	0.078	
8	EMP	0.147	0.151	0.011	0.009	-0.174	0.004	0.033

S3 Supplementary Material 3: Regression motivation

Fixed effect (FE) panel regression models are applied to analyze the statistical relation between the motifs and performance in industry-countries.

The rationale for using this type of model is that it allows us to explicitly control for unobserved (time-invariant) heterogeneity across industry-countries (such as norms and relative location within the EU or other aspects not captured by the controllers or by the definition of industries), which in itself may help reduce the impact of endogeneity. This is highly relevant in the European context due to the great variety of countries in terms of size, population, and economic structure which may influence the impact of new members differently. Moreover, owing to the within estimator that characterizes the fixed-effect model, it conditions how a change in motifs influences a change in value added over time. Compared to a pooled ordinary least squares (OLS) model where the between effect is emphasized (i.e., differences between cases), this approach emphasizes the dynamic relationship between our explanatory variables and the dependent variable over time.

The fixed-effect approach thus permits us to model changes in value added in one industry-country in relation to changes in motifs over time in that particular unit. A Hausman test comparing a random-effect model with the fixed effect model also confirms that the fixed-effect model is more efficient. We reduce the risk of reversed causality influencing the results by having all explanatory variables measured the year before the dependent variable. Apart from the case-specific fixed-effects, all models include a dummy capturing the recession 2008-2009, and cluster-robust standard errors at the country-level to control for the fact that the dynamics of economic activities within a given country might be more similar than between countries.

Since the dummy CEE is a time-invariant variable, it cannot be estimated within a FE-setting. Therefore, each model has been estimated in a two-step procedure. First, the main effects of each motif, Entry and the control variables are estimated to assess the general role of motifs and enlargement. Second, we include interaction effects between the motifs, Entry and CEE to explicitly assess whether changes in motifs in the CEE countries vs EU15 in relation to the enlargement influence performance.

Finally, all continuous variables are standardized (re-scaled to have a mean of zero and a standard deviation of one) to ease interpretation.

S4 Supplementary Material 4: Regression table

Table S4.4: Fixed effect panel regression models on the impact of Co-specialization network motifs and the entry of CEE countries in the EU on per capita value added per country-sector 2000-2014. Models 1-4 cover all industries and Models 5-8 are tested on separate industry sectors. Models 1-2 are on Overall motifs while the rest of the Models separate Internal and External motifs. Cluster-robust standard errors at country level within brackets. Significance at 10% (*), 5% (**) and 1% (***) confidence intervals.

	(1)	(2)	(3)	(4)	(5)	(6)	(7)	(8)
	All	All	All	All	Primary	Basic Manu	Capital Manu	Services
Main								
Overall	-0.020**	-0.018						
	(0.008)	(0.011)						
Internal			-0.027**	-0.015	-0.211*	0.228	-0.204*	-0.021
			(0.012)	(0.013)	(0.119)	(0.295)	(0.054)	(0.048)
External			-0.007	-0.012*	0.030	0.015	0.145*	-0.003
			(0.007)	(0.006)	(0.033)	(0.109)	(0.025)	(0.036)
Interactions								
CEE # Overall		-0.072**						
		(0.024)						
Entry # Overall		0.006						
		(0.007)						
CEE # Entry # Overall		0.078***						
		(0.023)						
CEE # Internal				-0.069*	0.137	-1.050*	0.281***	0.073
				(0.037)	(0.136)	(0.561)	(0.086)	(0.081)
Entry # Internal				-0.001	0.080	-0.087	0.035	0.008
				(0.010)	(0.173)	(0.280)	(0.099)	(0.026)
CEE # Entry # Internal				0.058**	-0.009	0.864*	-0.115	-0.129**
				(0.026)	(0.187)	(0.488)	(0.124)	(0.058)
CEE # External				-0.015	0.196	1.019	-0.278**	-0.115
				(0.018)	(0.193)	(0.665)	(0.120)	(0.081)
Entry # External				0.009	0.-0.017	0.016	-0.041	0.081
				(0.006)	(0.045)	(0.091)	(0.029)	(0.038)
CEE # Entry # External				0.026	-0.377	-1.064*	0.067	0.087
				(0.020)	(0.239)	(0.346)	(0.123)	(0.090)
Control variables								
Entry	0.113***	0.069***	0.112***	0.067**	0.239*	0.130	0.247**	0.094***
	(0.029)	(0.020)	(0.029)	(0.019)	(0.153)	(0.205)	(0.098)	(0.045)
Recession	-0.001	0.011	-0.000	0.011	-0.053***	0.069*	-0.080**	-0.009
	(0.029)	(0.028)	(0.029)	(0.028)	(0.030)	(0.081)	(0.056)	(0.028)
EMP	-0.287***	-0.254***	-0.280***	-0.241***	-0.029	0.458	0.278	-0.471***
	(0.048)	(0.041)	(0.047)	(0.041)	(0.377)	(0.310)	(0.570)	(0.203)
GFC	-0.028	-0.051**	-0.028	-0.053**	-0.070	-0.155*	-0.001	-0.018
	(0.019)	(0.016)	(0.019)	(0.017)	(0.050)	(0.088)	(0.063)	(0.026)
Intercept	-0.019	-0.039	-0.020	-0.036	-0.530***	0.261	-0.222**	0.440***
	(0.032)	(0.024)	(0.033)	(0.024)	(0.125)	(0.185)	(0.099)	(0.040)
Time trend	Y	Y	Y	Y	Y	Y	Y	Y
Industry-country FE	Y	Y	Y	Y	Y	Y	Y	Y
Year FE	Y	Y	Y	Y	Y	Y	Y	Y
R2 (within)	0.113	0.186	0.115	0.189	0.395	0.663	0.515	0.201
N (Groups)	5378	537	537	537	48	16	32	95
N (Observations)	8032	8032	8032	8032	720	240	480	1419
